# Mental Health Risk Detection From Social Media Text Data: A Scoping Review of the Machine Learning Research Landscape

**DOI:** 10.1002/pchj.70100

**Published:** 2026-05-15

**Authors:** Yiqing He, Yinning He, Darong Liu

**Affiliations:** ^1^ Department of Psychology Guangzhou University Guangzhou China; ^2^ Department of Artificial Intelligence, Faculty of Computer Science and Information Technology University of Malaya Kuala Lumpur Malaysia

**Keywords:** anxiety, depression, machine learning, mental health risk, social media

## Abstract

Machine learning approaches have been increasingly applied to social media text data for mental health risk detection. However, existing studies vary widely in target outcomes, data sources, labeling strategies, and evaluation practices, and a structured overview of recent research remains limited. This scoping review aims to map the recent research landscape of machine learning–based mental health risk detection using social media text data. Following PRISMA ScR guidelines, peer reviewed journal articles published between January 2021 and January 2026 were retrieved from PubMed, Web of Science, and IEEE Xplore. Studies applying machine learning or deep learning methods to social media text data for mental health risk detection were included and synthesized descriptively. A total of 136 studies were identified. Most focused on depression, anxiety, and suicide or self‐harm related risks. Mental health risk was predominantly operationalized through proxy indicators derived from user‐generated content, with limited use of survey‐linked or clinically anchored labels. Traditional machine learning, deep learning, and Transformer‐based models coexisted, alongside substantial heterogeneity in validation strategies and performance metrics. Current research primarily targets proxy‐based mental health risk signals rather than clinical diagnoses. This review clarifies prevailing research emphases and methodological practices, and supports the use of social media–based approaches for population‐level monitoring and early risk identification.

## 1 Introduction

Mental health problems have become a major issue in global public health (Moitra et al. 2023). Psychological disorders such as depression, anxiety, and suicide risk have shown a sustained upward trend worldwide and have placed substantial pressure on individual well‐being, social functioning, and healthcare systems (Kaminsky et al. 2024; Pierce et al. 2024; Zhang et al. 2024). Traditional mental health detection and assessment mainly rely on clinical interviews, self‐report questionnaires, and face‐to‐face diagnosis (Mirea et al. 2021), yet clinical screening often struggles to capture dynamic changes in individuals'emotional states in a timely manner and to reach potential high‐risk populations who have not actively sought help or entered the healthcare system, resulting in certain limitations in coverage, temporal sensitivity, and ecological validity.

In the context of the widespread adoption of the internet and social media, individuals' emotional expressions, lived experiences, and social interactions are increasingly presented through online platforms, forming user generated content with real time and contextual characteristics, mainly including textual expressions and their accompanying interaction features such as posting frequency, topic participation, and language use patterns (Xu et al. 2024). Large scale textual data generated by social media platforms such as Twitter, Reddit, and Weibo are considered to contain rich psychological cues, including emotional vocabulary, thematic focus, and narrative style, and are therefore increasingly regarded as a valuable complement to traditional psychometric methods (Kim et al. 2023; Wang and Zhao 2022; Zarate et al. 2023). This trend has promoted the development of interdisciplinary research on mental health risk detection across fields such as computational social science, psychoinformatics, and natural language processing.

In recent years, machine learning and deep learning techniques have made rapid advances in tasks such as text classification, sentiment analysis, and risk prediction, providing an important methodological foundation for data driven mental health monitoring based on social media text data. Researchers have widely adopted methods such as support vector machines (SVM), random forests (RF), convolutional neural networks (CNN), long short term memory networks (LSTM), and Transformer based pretrained models such as bidirectional encoder representations from Transformers (BERT) to identify language patterns and expressive features associated with depression, anxiety, suicide risk, and other psychological distress (Abbas et al. 2024; Baydili et al. 2025; Xin and Zakaria 2024).

Existing empirical studies indicate that approaches based on natural language processing (NLP) can extract linguistic features related to emotional experiences and psychological distress from social media texts and support mental state identification and risk detection. Kabir et al. (2022) used NLP methods to annotate user texts with emotion‐related labels and achieved an accuracy of approximately 81% in anxiety and depression detection tasks, demonstrating the feasibility of such approaches in practical detection scenarios. Researchers have also attempted to model users' expression trajectories and risk change processes on social media through deep learning models. Kangshun et al. (2023) applied long short‐term memory networks (LSTM) to the sequential modeling of Weibo users' posting behaviors to identify potential high‐risk populations, further highlighting the application potential of sequential methods in early warning. Helmy et al. (2024) applied sentiment analysis models to Twitter data for emotional state classification, achieving an accuracy of 92% in binary classification tasks and approximately 88% in multiclass tasks. In text scenarios containing negation expressions, the F1 score remained between 85% and 87%, suggesting that affective computing models exhibit a certain degree of robustness under complex linguistic phenomena. Recent studies have also begun to explore the adaptability and extensibility of pretrained models based on the Transformer architecture in this field (Yang et al. 2025), but the overall research landscape still shows substantial heterogeneity in methods and tasks. As conceptualized in recent literature, mental health risk detection often encompasses a broad spectrum of tasks, ranging from distress‐related expression identification to proxy‐based vulnerability estimation, rather than strict prediction of incident clinical outcomes.

Nevertheless, existing review studies have mainly focused on the associations between social media text data and mental health states, such as risk signal identification and high risk early warning, and typically concentrate on specific mental health topics (Ahmed et al. 2022), specific social media platforms (Hussain et al. 2020), or a single algorithmic paradigm (Tahir et al. 2025), thereby providing valuable summaries of methodological feasibility, performance ranges, and technical pathways to some extent. However, systematic efforts to map the research structure and methodological landscape of this field from an integrative perspective that spans multiple mental health outcomes, cross‐platform data sources, and diverse machine learning approaches remain relatively limited. In addition, substantial differences across studies in model selection, evaluation metrics, and data scale further reduce the possibility of conducting comprehensive comparisons and achieving an overarching understanding of the overall research ecosystem.

Based on the above research background and identified gaps, this study adopts a scoping review approach to systematically examine studies published from 2021 to early 2026 that use social media text data and machine learning methods for mental health risk detection. This review aims to address the following questions: (1) What temporal trends and geographic distribution patterns have emerged in this field in recent years? (2) What types of mental health risk outcomes are primarily examined in existing studies? (3) What types of machine learning and deep learning models have been applied in social media–based mental health risk detection, and how are these methods distributed across data sources and study contexts? (4) Which metrics are typically used to evaluate model performance? By systematically integrating this information, the study seeks to construct an overall knowledge map of the field, identify research hotspots and structural gaps, and provide methodological references for future research and public health practice.

## 2 Methods

### 2.1 Review Framework

This study adopts a scoping review approach to examine the research structure and trends of mental health risk detection based on social media text data. The review process follows the Preferred Reporting Items for Systematic Reviews and Meta Analyses extension for Scoping Reviews (PRISMA ScR) guidelines (Tricco et al. 2018). In the context of social media research, mental health risk is often operationalized through proxy indicators, including sustained negative affect, emotion dysregulation, or distress‐related linguistic patterns, rather than direct clinical diagnosis.

### 2.2 Search Strategy

To systematically retrieve relevant literature, this study selected three databases: PubMed, Web of Science, and IEEE. The search date was January 16, 2026, and the search language was restricted to English. The search query was (“Social Media” OR “Twitter” OR “Reddit” OR “Weibo”) AND (“Machine Learning” OR “Deep Learning” OR “NLP” OR “Transformer” OR “BERT”) AND (“Mental Health” OR “Depression” OR “Anxiety” OR “Suicide Risk”).

### 2.3 Inclusion and Exclusion Criteria

To be included, studies were required to propose a machine learning–based method or technique primarily aimed at detecting mental health risk through social media platforms such as Weibo, Reddit, or Twitter. The review focused on studies using social media text data or text‐centered user‐generated content, as textual data represent the most common and methodologically comparable evidence base in this field. Behavioral and interactional indicators were not treated as an independent inclusion domain. This review included peer‐reviewed studies published in English between January 1, 2021 and January 16, 2026 and indexed in PubMed, Web of Science, and IEEE. Reviews, conference abstracts, proposals, editorials, conference proceedings, master's and doctoral theses, reports, and purely qualitative studies were excluded. IEEE Xplore was included to capture journal articles indexed in engineering and computational science domains, and only peer‐reviewed journal articles were retained. Journal articles were identified based on document type and journal indexing information provided by IEEE Xplore. Conference proceedings were excluded to ensure methodological completeness and reproducibility of reported machine learning pipelines. The inclusion and exclusion criteria of this review are shown in Table 1.

**TABLE 1:** Inclusion and exclusion criteria.

Criteria	Specified criteria
Inclusion	Studies using social media text data or text‐centered user‐generated content to detect mental health risk Studies employing machine learning or deep learning models Studies using user generated content and focusing on prediction or classification models Peer reviewed journal articles Published between January 1, 2021 and January 16, 2026 Published in English
Exclusion	Studies that do not specify the source of social media text datasets Studies relying solely on manual coding or annotation without any machine learning–based modeling, prediction, or validation pipeline Studies lacking performance metrics or methodological details Non peer reviewed publications such as conference abstracts, editorials, reviews, reports, master's or doctoral theses, and purely qualitative studies

### 2.4 Study Selection

The literature screening process of this review was conducted in three stages. First, the first reviewer removed duplicates and downloaded full texts. Second, two reviewers independently screened titles and abstracts to determine eligibility according to the inclusion criteria. Third, the two reviewers independently assessed full texts and finalized the included studies. During the screening process, any disagreements between the two reviewers were resolved through discussion and consensus, with a third researcher involved when necessary. The screening procedure followed the PRISMA ScR guidelines, and the results are presented in Section 3.1 Search Results. To assess the consistency of the screening process, Cohen's Kappa coefficient was calculated at the title and abstract screening stage to evaluate inter‐rater reliability between the two reviewers.

### 2.5 Data Extraction and Synthesis

A data extraction form was developed and pilot tested on three randomly selected included studies to refine field definitions and ensure consistency see Supporting Infomration 1. Two reviewers independently used Excel worksheets to extract and identify data related to study characteristics, predictive models used, and training data sources. The extracted data were synthesized using a narrative approach. Studies were categorized according to their research focus, and mental health outcomes were grouped into five thematic categories see Table 2, with suicide and self‐harm treated as a separate risk‐oriented category and non‐diagnostic distress and general well‐being listed separately. We systematically recorded the machine learning prediction models reported in the studies such as linear regression (LR), support vector machines (SVM), and other machine learning algorithms and deep learning models, the data sources used for training such as Reddit and Twitter, the sample size of the data such as the number of posts analyzed, the types of mental health problems examined such as depression and anxiety, the performance evaluation metrics such as accuracy and F1 score, and the best detection performance achieved by the models. During the data extraction stage, the two reviewers independently extracted data and conducted a consistency check on 10% of the sample, with inter‐rater reliability calculated using Cohen's Kappa.

**TABLE 2:** Classification of nominal mental health targets used as risk proxies (*n* = 136).

Mental health categories	Representative labels	Number of studies (%)
Common mental health problems	Depression Anxiety Depression + Anxiety Interaction anxiousness Post‐traumatic stress disorder Depression + Stress (excluding suicide)	63 (46.3%)
Suicide and self‐harm outcomes	Suicide Self‐harm Depression + Suicide Multiple (suicide/self‐harm)	38 (27.9%)
Severe/complex psychiatric conditions	Schizophrenia Anorexia nervosa Bipolar disorder Neuropsychiatric symptoms Multiple (Severe Mental Disorders)	11 (8.2%)
Psychological distress states (non‐diagnostic)	Emotional distress Emotional contagion Mental distress Psychological distress Occupational emotional distress Psychosocial stressors Stress	12 (8.8%)
General mental health and well‐being	Need for affect Emotional well‐being Mental health resilience Psychological/subjective well‐being General emotional sentiment Public emotional response/sentiment General mental health Emotion/affective state	12 (8.8%)

## 3 Results

### 3.1 Search Results

**FIGURE 1:**
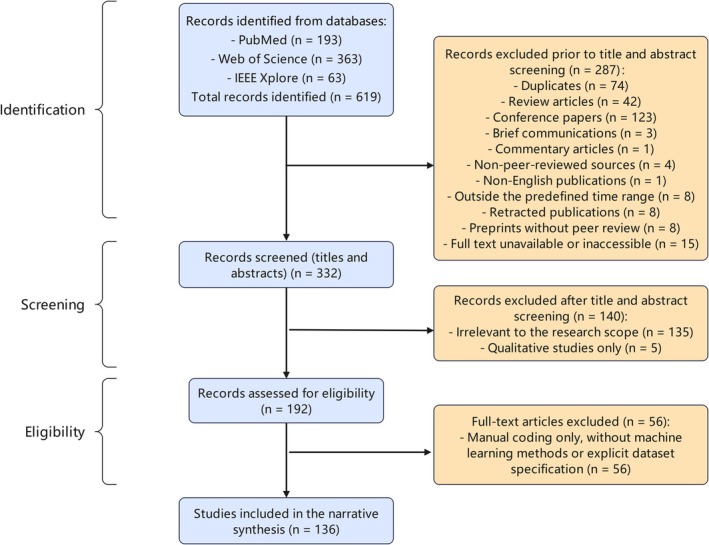
PRISMA chart.

According to the screening criteria of this review, the study inclusion process is illustrated in Figure 1. A total of 619 records were retrieved from the three databases: PubMed, Web of Science, and IEEE. After deduplication, records that were reviews, conference‐related items, non‐peer‐reviewed document types, publications outside the target years or languages, retracted or preprint records, and studies with inaccessible full texts were excluded, leaving 332 articles for title and abstract screening.

During the title and abstract screening stage, 135 records that were not relevant to the topic and five qualitative studies that did not include reproducible machine learning modeling and performance evaluation information were removed, resulting in 192 articles entering the full text screening stage.

At the full text screening stage, a further 56 articles were excluded, mainly because they did not specify the social media text data source, did not involve machine learning models, lacked performance metrics or key methodological details, or relied solely on manual coding or annotation without constructing machine learning models. Ultimately, 136 articles were included in this review, as detailed in Supporting Information 2. Inter‐rater agreement between the two reviewers was high at the title and abstract screening stage with Cohen's Kappa of 0.78, and consistency was also high at the data extraction stage with Cohen's Kappa of 0.82.

### 3.2 Characteristics of Included Studies

**FIGURE 2:**
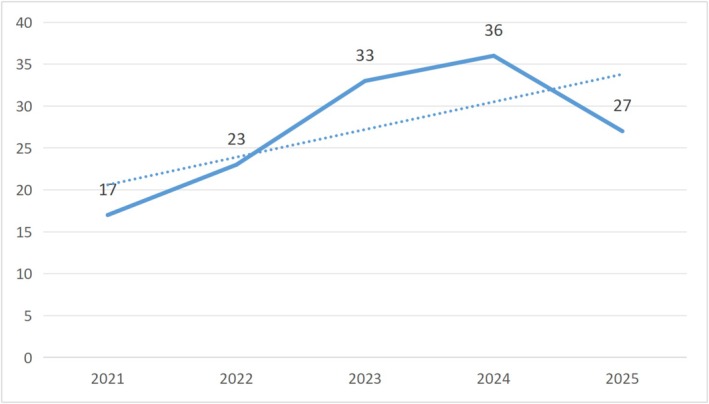
Trend in the number of publications (*n* = 136). The search was conducted up to January 16, 2026. However, no eligible studies published in 2026 met the inclusion criteria after screening; therefore, the annual trend is presented for the complete years 2021–2025 only.

Between 2021 and 2025, the number of studies in this field showed a clear overall upward trend. Annual publication output increased from 17 articles in 2021 to 36 articles in 2024, indicating sustained academic attention to this research topic in recent years and a relatively active phase during 2023 and 2024. Although the number of publications in 2025 declined compared with the previous year at 27 articles, it remained higher than in the earlier years, suggesting that research activity in this field has generally been maintained at a relatively stable and high level see Figure 2.

**FIGURE 3:**
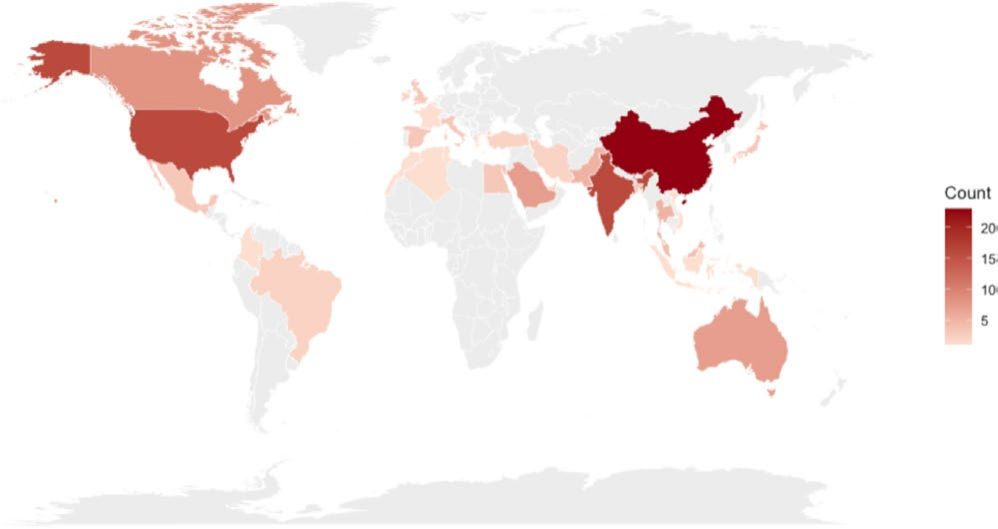
Global distribution of related studies (*n* = 136).

In terms of geographic distribution, related studies exhibited clear cross‐regional characteristics, with research outputs spanning Asia, North America, Europe, Oceania, as well as parts of Africa and Latin America. Asia was the primary source of research output in this field, with China producing 23 articles and India 16 articles, followed by Saudi Arabia and Australia with seven articles each, South Korea, Pakistan, and Thailand with five articles each, and Malaysia with four articles. In North America, the United States, with 16 articles, and Canada, with eight articles, constituted the main contributors. European research output was relatively dispersed, with the United Kingdom, Italy, and Spain each contributing three articles, while other countries produced fewer publications. A small number of studies also originated from Africa and Latin America, indicating that this research topic has a certain degree of global coverage; see Figure 3.

### 3.3 Mental Health Conditions

Among the 136 included studies, research targets were primarily concentrated on common mental health problems. Mental health outcomes were categorized based on the nominal targets reported in each study, regardless of whether labels reflected clinically confirmed diagnoses or proxy‐based risk indicators. Specifically, common mental health problems constituted the largest category, accounting for 63 studies or 46.3%, mainly involving depression, anxiety, comorbid depression and anxiety, interaction anxiousness, posttraumatic stress disorder (PTSD), and co‐occurring depression and stress states without suicide outcomes.

Second, suicide and self‐harm outcomes were a major focus in 38 studies or 27.9%, covering suicide, self‐harm behaviors, comorbid depression and suicide, and multi‐label outcomes including suicide or self‐harm, reflecting the strong risk detection and early warning orientation of social media‐based mental health research.

By contrast, psychological distress states accounted for a smaller proportion with 12 studies or 8.8%, primarily including emotional distress, psychological stress, psychosocial stressors, emotional contagion, and work‐related emotional distress, emphasizing psychological reactions or process‐oriented states rather than explicit diagnoses. At the same time, studies on general mental health and well‐being were equally represented with 12 studies or 8.8%, mostly examining overall mental health, emotional well‐being, psychological resilience, or collective emotional responses from a broader perspective.

In addition, research on severe or complex psychiatric conditions was relatively limited, comprising only 11 studies or 8.2%, mainly addressing schizophrenia, bipolar disorder, neuropsychiatric symptoms, anorexia nervosa, and multiple comorbid conditions. These studies primarily focused on identifying disorder‐related linguistic patterns that served as proxy indicators of psychological vulnerability, rather than as direct markers of clinical conditions. This distinction underscores the conceptual boundary between risk detection and condition recognition.

Overall, research in this field continues to focus predominantly on affective disorders and suicide or self‐harm risk, while relatively limited attention has been paid to severe or complex psychiatric conditions and broader mental health risk profiles see Table 2.

### 3.4 Mental Health Labeling Criteria

Among the included studies, the most common labeling approach was community or platform‐based proxy labels, used in 104 studies and accounting for approximately 76.5% of the total sample. Such studies typically relied on structural features of social media platforms, such as specific topic communities, keywords, hashtags, or user self‐descriptions, and inferred individual mental health risk through rule‐based matching or weakly supervised, model‐driven labeling strategies rather than individual‐level clinical diagnosis.

Second, hybrid labeling strategies combining machine learning and manual identification were adopted in 19 studies or 14.0%, in which automated model‐based screening was integrated with human identification or correction to improve label accuracy and reliability.

By comparison, survey based clinical or self report measurement approaches accounted for a relatively small proportion, with only seven studies or 5.1% using questionnaires or self report measures to assess mental health status. Studies that directly relied on clinical or administrative service records as labeling sources were the least common, with only six studies or 4.4%.

**FIGURE 4:**
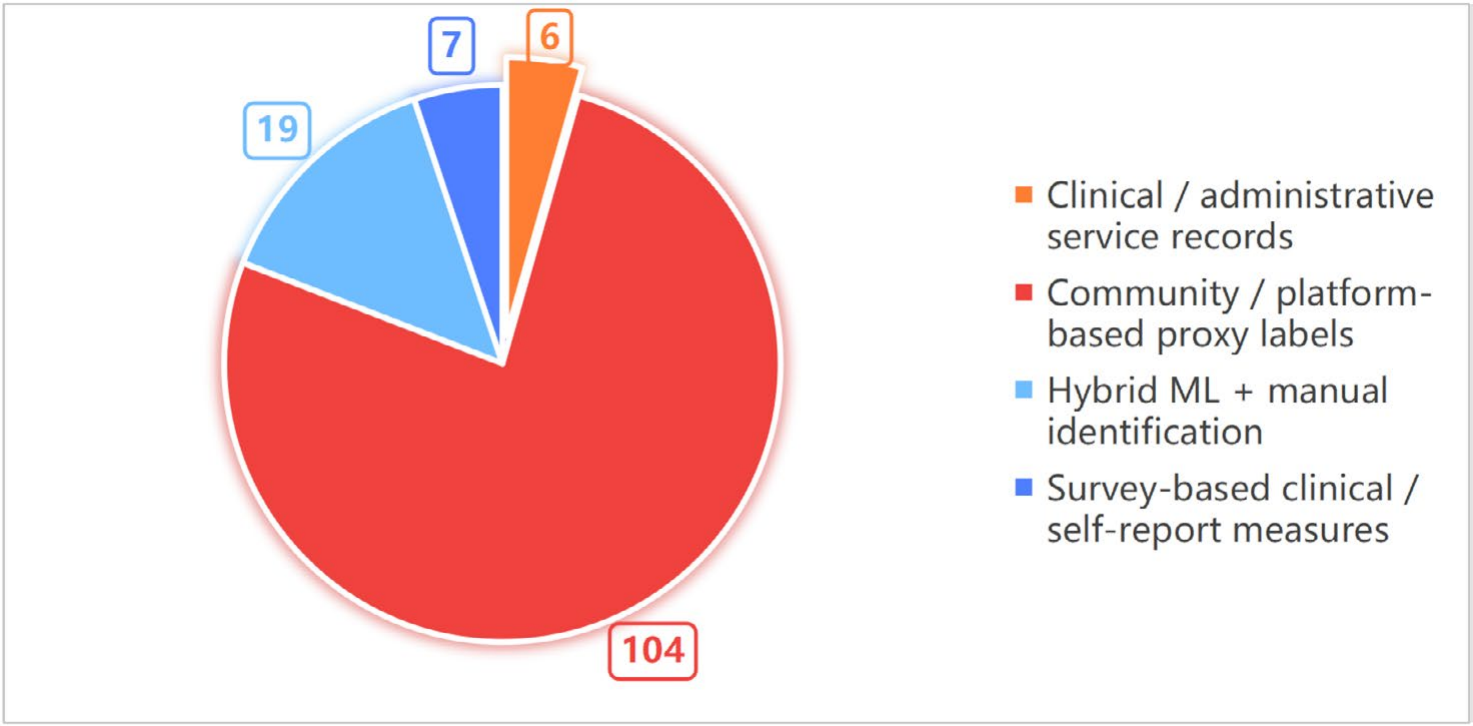
Distribution of data labeling strategies in social media‐based mental health studies (*n* = 136). Manual identification was considered acceptable only when embedded within a machine learning–based modeling or validation pipeline.

Overall, existing research has shown a strong tendency to rely on platform level proxy labels for mental health risk identification, while the use of real clinical data remains limited,‐ see Figure 4.

### 3.5 Social Media Text Data Sources and Machine Learning Models

Table 3 summarizes the social media text data sources and corresponding machine learning modeling methods used in the studies included in this scoping review. Overall, research has primarily relied on a small number of major social media platforms, with Twitter being the most commonly used data source with 63 studies, followed by Reddit with 39 studies and Weibo with 16 studies. Some studies integrated data from multiple platforms, most commonly combining Twitter and Reddit with 16 studies, while other multi‐platform combinations such as Facebook with Twitter and Reddit or Douban with Zhihu and Weibo were rare and appeared only in a small number of studies. In general, existing research shows a clear preference for platforms with high openness, large scale textual data, and ease of access as data sources.

**TABLE 3:** social media text data sources, predictive models, and performance metrics in the reviewed studies (*n* = 136).

Predictive models and their primary metrics observed in reviewed studies																
Data type	No.	Predictive model used														
		AdaBoost	CNN	GRU	KNN	MultiLinReg	LinReg	LogReg	TopicModel	LSTM	MLP	NB	RF	DT	SVM	BERT
Twitter	63	Y	Y	Y	Y		Y	Y	Y	Y	Y	Y	Y	Y	Y	Y
Reddit	39		Y	Y	Y			Y	Y	Y		Y	Y	Y	Y	Y
Weibo	16	Y	Y			Y	Y	Y	Y	Y		Y	Y		Y	Y
Reddit + Twitter	16		Y	Y				Y		Y		Y	Y		Y	Y
Facebook + Twitter + Reddit	1			Y				Y					Y		Y	Y
Douban + Zhihu + Weibo	1									Y						Y

Predictive models and their primary metrics observed in reviewed studies															
Data type	No.	Predictive model used									Primary performance metrics	Performance range (for data type) min–max	Language of data samples analyzed	Data volume/users (range)	References
		BERT	XGBOOST	Transformer	LASSO	LDA	K‐means	LLMs	Word2Vec	Others					
Twitter	63	Y	Y		Y	Y			Y	Latent Semantic Scaling, XLNet, GloVe, LPA, LDA, RNN	Precision: 29	0.13–0.99	English–55	377~471,553,966	S01, S06, S09, S10, S12, S15, S16, S20, S26, S32, S34, S36, S40, S41, S42, S50, S51, S52, S54, S57, S58, S61, S62, S63, S64, S67, S68, S70, S71, S72, S73, S75, S76, S77, S80, S82, S84, S85, S86, S87, S88, S90, S91, S92, S93, S98, S99, S100, S106, S107, S108, S110, S115, S116, S117, S118, S120, S124, S125, S128, S130, S131, S135
											Recall: 28	0.32–0.96	Japanese–4	2432~62,000,000	
											Accuracy: 39	0.4–0.99	Spanish–3	503~28268	
											AUC: 8	0.49–0.93	Thai–4 Arabic–3 Korean–2 Russian–1 Deutsch–1 French–1 Portuguese–1		
											F1 Score: 39	0.39–0.96			
											LSS scores: 1	0.219			
											Cohen d: 2	0.33–0.44			
											Cohen's Kappa: 2	0.79–0.85			
											Negative sentiment proportion: 1	0.28–0.47			
Reddit	39	Y	Y	Y	Y	Y	Y	Y	Y	SARIMA, SAGE, LightGBM	F1 Score: 31	0.43–0.95	English–39	2021~2,100,000	S02, S07, S13, S17, S19, S23, S25, S27, S28, S31, S35, S39, S43, S53, S55, S56, S60, S66, S81, S83, S89, S94, S95, S96, S102, S103, S104, S109, S111, S112, S114, S119, S122, S123, S127, S129, S132, S133, S136
											Recall: 14	0.74–0.98			
											Accuracy: 19	0.75–0.98			
											Precision: 14	0.74~0.94			
											Pearson's r: 1	0.73–0.86			
											SMAPE: 1	13.3			
											Cohen d: 2	0.81			
											AUC: 5	0.78			
Weibo	16	Y	Y	Y				Y	Y	Transformer, TreeReg, GBR, GBDT	F1 Score: 9	0.68–0.97	Chinese–16	300~895,012	S03, S04, S05, S08, S11, S14, S22, S29, S30, S33, S59, S69, S79, S101, S113, S126
											Precision: 5	0.68–0.95			
											Recall: 5	0.67–0.95			
											Accuracy: 6	0.86–0.95			
											AUC: 3	0.82–0.93			
											Correlation (r): 4	0.32–0.65			
											Criterion validity: 1	0.3			
											Split‐half reliability: 1	0.755			
											Adjusted R: 1	0.42			
Reddit + Twitter	16	Y	Y	Y				Y	Y		Proximity‐based score: 1	0.28	English–17	2,882~658,177	S24, S37, S38, S44, S45, S46, S47, S48, S49, S65, S74, S78, S97, S105, S121, S134
											F1 Score: 10	0.84–0.97			
											Accuracy: 10	0.85–0.99			
Facebook + Twitter + Reddit	1	Y								LightGBM	AUC: 1	0.97–0.99	English–1	52,681	S21
											F1 Score: 1	0.93–0.96			
Douban + Zhihu + Weibo	1	Y				Y				CRF	Accuracy: 1	0.92	Chinese–1	966,746	S18

*Note:* The studies summarized in Table 3 are labeled as S01–S136, with complete bibliographic information provided in Supporting Information: 2. By contrast, the reference list in the main manuscript contains only the works cited directly in the text, so the numbers are not intended to correspond one‐to‐one. Counts for languages and metrics are not mutually exclusive, as some studies used multiple languages and/or reported multiple evaluation metrics. Performance ranges are reported as the minimum and maximum values observed across different studies and tasks within each data source, rather than within a single model or dataset. English‐only refers to publication language; the language field in Table 3 indicates the language of the social media text data analyzed.

In terms of predictive models, the included studies adopted a wide range of traditional machine learning and deep learning methods. Support vector machines and random forests remained the most commonly used traditional machine learning models and were frequently applied across studies using Twitter, Reddit, and Weibo data. At the same time, classical algorithms such as naive Bayes, k nearest neighbors, and logistic regression appeared in a substantial number of studies. With the development of the field, the use of deep learning models has increased markedly, with convolutional neural networks and long short term memory networks being widely applied to data from different platforms. GRU, multilayer perceptrons, XGBoost, and Transformer‐based models such as BERT and XLNet have also increasingly appeared in recent studies. A small number of studies further introduced large language models or task‐specific customized algorithms, reflecting a gradual expansion of the field toward more complex representation learning and generative modeling approaches. Overall, traditional machine learning methods still dominate current research, but the growing application of deep learning and pretrained models indicates an increasingly diversified methodological landscape.

Regarding model performance evaluation, almost all studies reported at least one quantitative metric. Accuracy and F1 score were the most commonly used primary performance indicators and were frequently reported in studies based on Twitter and Reddit data. Precision and recall also appeared as supplementary metrics in a considerable number of studies. By contrast, AUC, Cohen's Kappa, correlation coefficients, and affect proportion measures were used in only a small number of studies, indicating variability in the choice of evaluation metrics across research. Performance ranges also differed markedly by data source, with models based on Twitter and Reddit data showing wide performance distributions, while studies based on Weibo data generally fluctuated within the medium to high performance range. These performance ranges should be interpreted descriptively rather than comparatively, given substantial heterogeneity in tasks, labels, and data sources.

In terms of language use, the research samples were predominantly based on English language data, especially in studies related to Twitter and Reddit, where English data held a clear dominance. Chinese language data were mainly concentrated in studies using Weibo and its combined platforms. A small number of studies involved other languages such as Arabic, Spanish, Japanese, Korean, and Russian, and a few studies adopted multilingual data for analysis. With respect to sample size, the scale of participants or textual data varied considerably across studies, with the smallest samples including approximately 300 instances and the largest reaching hundreds of millions, demonstrating the substantial variability in data volume characteristic of social media‐based mental health research.

## 4 Discussion

### 4.1 Key Findings and Technical Considerations

This scoping review synthesizes machine learning studies that detect mental health risk signals from social media text data. The included literature suggests that the field has largely operationalized “risk” as proxy indicators captured in user generated content, such as distress related expressions, self disclosed symptoms, and vulnerability linked linguistic patterns, rather than confirmed clinical incidence. Studies that explicitly framed their objectives as risk signal identification or early warning, instead of diagnosis, were common across platforms (S01, S11, S13).

Many studies focused on common mental health problems such as depression and anxiety and on suicide or self‐harm related outcomes, which tend to have more observable expression markers in social media discourse (S05, S33, S82, S114). A smaller subset addressed severe or complex psychiatric conditions, for example schizophrenia or anorexia nervosa, often emphasizing disorder‐related linguistic correlates rather than diagnostic validity (S17, S32). This outcome concentration has practical implications for model interpretation. Even when predictive performance appears strong, it primarily reflects the separability of proxy labeled groups under specific platform contexts, not clinical diagnostic accuracy.

In addition, many studies relied on platform or community‐based proxy labels, including community membership and weak supervision strategies (S11, S19, S23). In contrast, a limited group linked labels to survey or self‐report measurements, which improves construct alignment but typically reduces scale and introduces sampling constraints (S6). A few studies further attempted stronger validation designs, such as independent algorithm validation or transfer learning to clinically adjacent text, which helps narrow the gap between benchmark‐style modeling and applied settings (S01, S25).

With respect to modeling choices, traditional machine learning models remain widely used, alongside deep learning and Transformer‐based approaches. Transformer and enhanced BERT‐style models were frequently adopted for contextual representation, including cross‐language or multilingual settings (S50, S64, S77). Some studies evaluated large language models or combined them with ensembles for monitoring, indicating a recent methodological expansion, but the evidence base is still uneven across tasks and platforms (S37, S51, S81). Importantly, model complexity does not automatically translate into better real‐world utility. Several papers explicitly compared classical and deep approaches and emphasized trade‐offs related to data requirements, robustness, and interpretability (S21, S37).

Evaluation practices also varied substantially. Accuracy and F1 score were reported most often, while calibration, external validation, and temporally robust testing were less consistently addressed. A notable positive signal is that a small number of studies incorporated calibration or related reliability considerations for Transformer based models, which is highly relevant for risk oriented applications (S123). Taken together, these observations support interpreting reported performance descriptively within each study context. Cross study comparisons remain inappropriate unless task definitions, label construction, and validation protocols are aligned.

### 4.2 Ethical and Clinical Implications

Ethically, the dominance of proxy labeling implies a nontrivial risk of misclassification and over‐inference, as community‐based labeling and self‐disclosure heuristics may conflate expressions of distress with the presence of mental disorders. Such conflation can amplify stigma when model outputs are interpreted as diagnostic claims. In practical terms, false positive classifications may lead to unnecessary labeling, anxiety, or social consequences for individuals, whereas false negatives may delay the recognition of serious risk and impede timely support, particularly in suicide‐related contexts. Against this backdrop, studies that explicitly distinguish risk signal identification from diagnosis provide an important framing for responsible use, especially in high‐risk monitoring scenarios (S13, S16). This distinction should therefore be preserved in how results are communicated to stakeholders and how systems are deployed.

From a clinical translation perspective, the reviewed evidence highlights substantial practical constraints on the direct application of social media–based risk detection models. Most studies do not specify clear accountability structures or intervention protocols following algorithmic risk identification, and there is currently no established clinical pathway for integrating such signals into routine mental health care. In light of these constraints, the reviewed evidence supports a cautious positioning of these models as screening and surveillance aids rather than stand‐alone diagnostic tools. Approaches that connect social media signals with downstream service use or clinically adjacent communication illustrate a more realistic translational pathway, in which model outputs inform resource planning or triage rather than replace clinical assessment (S2, S25). For public health practice, model outputs may therefore be most defensible at aggregated levels, such as community‐level trend monitoring or early warning, rather than individual‐level labeling.

At a broader structural level, evidence from disorder‐specific tasks indicates that model outputs can reflect social and cultural biases embedded in platform participation and language norms. For example, gender bias considerations raised in anorexia‐related modeling suggest that model behavior may vary across demographic groups and reinforce harmful stereotypes if left unaudited (S32). These concerns extend beyond specific disorders, as the predominant reliance on English‐language data and platform‐specific populations raises broader questions about the transferability of models across linguistic, cultural, and socio‐economic contexts, with potential implications for health equity. In this context, increasing model sophistication, particularly through the use of pretrained models and large language models, makes explainability and accountability increasingly critical. Studies adopting explainable frameworks for high‐risk outcomes provide an initial direction for improving transparency and governance, but such efforts remain limited relative to the overall literature base (S43).

### 4.3 Limitations and Future Researches

In terms of data sources, the evidence base synthesized in this review is shaped by both platform ecology and accessibility constraints. The included studies relied predominantly on high availability text platforms such as Twitter and Reddit, while Weibo and multi platform datasets were comparatively less frequent. This concentration may limit cultural and contextual generalizability, especially when linguistic norms, self disclosure practices, and platform governance differ across regions. Although multilingual or non English studies were present, English data still dominated the reviewed pipelines, and non English datasets were often smaller or more heterogeneous in sampling and labeling, which may affect cross cultural transferability. In addition, most studies remained text centric, with fewer incorporating non textual or interaction level signals, suggesting that current risk detection models may capture only part of the behavioral context available in social media environments. Accordingly, the present review primarily synthesized text‐based studies and did not treat behavioral or interactional indicators as a separate inclusion domain. Future reviews may provide a more comprehensive synthesis by systematically incorporating non‐textual dimensions, such as behavioral and interactional indicators, into the review framework. Future research can expand cross platform and cross language validation, and explore multimodal designs that integrate text with behavioral and interaction features, while maintaining transparent data provenance and privacy safeguards. Such efforts may include fusing textual content with image‐based cues or interaction patterns, as well as modeling temporal dynamics through longitudinal or network‐based representations, which introduce both opportunities for richer context modeling and challenges related to data alignment, interpretability, and privacy. Evidence from multi platform or multimodal efforts in the included literature indicates feasibility but also highlights the need for systematic benchmarking across heterogeneous data environments.

Methodologically, several design choices in this scoping review necessarily define its coverage boundary and should be considered when interpreting the landscape. First, the search window was restricted to 2021 to early 2026 to capture the period of rapid growth and reflect contemporary methodological practice, but this decision may have excluded earlier exploratory studies that shaped foundational task formulations and labeling conventions. Second, the search was conducted in IEEE Xplore, Web of Science, and PubMed and was limited to English language publications. As a result, relevant studies indexed in other databases or published in other languages may have been missed, which may introduce database and language‐related selection bias. Third, a small proportion of records could not be assessed at the full‐text level due to the unavailability of the complete articles. This constitutes a potential availability bias and may have marginally constrained the comprehensiveness and representativeness of the included evidence. Beyond these review‐level constraints, the reviewed primary studies also exhibited substantial methodological heterogeneity in proxy labeling, validation protocols, and reporting practices. Many models learn to separate proxy‐defined groups rather than clinically verified outcomes, and external validation, temporal robustness checks, and calibration were not consistently implemented, although a subset of studies provides useful exemplars such as independent validation, transfer to clinically adjacent text, and calibration‐oriented evaluation. Future research should strengthen construct alignment by clarifying what “risk” represents in each task, standardize reporting for labels and evaluation metrics, and adopt stronger validation hierarchies including cross‐platform testing, temporally separated evaluation, and independent replication.

## 5 Conclusion

This scoping review provides a structured overview of machine learning studies that detect mental health risk from social media text data. Across the reviewed literature, research predominantly targets depression, anxiety, and suicide or self‐harm related risks, and operationalizes mental health risk through proxy indicators derived from user‐generated content rather than clinical diagnoses. Platform or community‐based labeling is the most common strategy, while clinically anchored validation remains limited.

By synthesizing evidence from 136 studies, this review clarifies current research emphases and methodological practices in the field. The findings underscore the role of social media–based machine learning approaches as tools for population‐level monitoring and early risk identification, and offer a reference framework for future research aiming to improve conceptual clarity, validation rigor, and responsible application in mental health and public health contexts.

## Funding

This research was supported by grants from Guangdong Provincial Philosophy and Social Science Planning Project (GD23XXW11) and Guangdong Provincial Department of Education Innovation‐Driven University Strengthening Program for Youth Innovation Talent (2024WQNCX072).

## Ethics Statement

The authors have nothing to report.

## Consent

The authors have nothing to report.

## Conflicts of Interest

The authors declare no conflicts of interest.

## Supporting information


**Data S1:** Data extraction form.

**Data S2:** pchj70100‐sup‐0002‐Supinfo2.xlsx.

## Data Availability

The data that supports the findings of this study are available in the Supporting Information of this article.
