# Supplementary material for: Mental Health Risk Detection From Social Media Text Data: A Scoping Review of the Machine Learning Research Landscape
Source: Psych J. 2026 May 15;15(3):e70100. doi: 10.1002/pchj.70100 (PMC13176780; doi:10.1002/pchj.70100)
Supplement: Supplementary file 1 — Data S1: Data extraction form. [file PCHJ-15-e70100-s002.docx]

## Supplementary 1. Data extraction form

| **Concept** | **Operational definition / Extracted information** |
| --- | --- |
| **Study characteristics** |  |
| Author | First author of the included study. |
| Year of publication | Year in which the study was published. |
| Country of publication | Country where the first author is affiliated. |
| Publication type | Type of publication (e.g., peer-reviewed journal article, conference article, master’s thesis, doctoral dissertation). |
| **Social media data characteristics** |  |
| Social media platform(s) | Social media platform(s) from which data were collected (e.g., Twitter, Reddit, Weibo, or multi-platform combinations). |
| Data modality | Type of user-generated text content analyzed (e.g., posts, tweets, comments, replies). |
| Language of data samples | Language(s) of the social media data analyzed (e.g., English, Chinese, Arabic). |
| Dataset size | Total number of text units used for analysis (e.g., number of posts, tweets, or comments). |
| Number of users | Total number of unique users whose social media data were included in the study, if reported. |
| Time span of data | Time period during which the social media data were collected, if specified. |
| **Mental health identification** |  |
| Mental health outcome(s) | Type(s) of mental health conditions or outcomes investigated (e.g., depression, anxiety, suicide, psychological distress), coded according to the classification framework in Table 2. |
| Labeling strategy | Method used to identify mental health status, including community/platform-based proxy labels, hybrid ML + manual identification, survey-based self-report or clinical measures, or clinical/administrative records. |
| Assessment instruments (if any) | Standardized scales or questionnaires used for labeling or validation (e.g., PHQ-9, CES-D), if applicable. |
| **AI and modeling characteristics** |  |
| Machine learning / AI model(s) | Predictive models or algorithms used (e.g., SVM, Random Forest, Naïve Bayes, CNN, LSTM, Transformer-based models, LLMs). |
| Feature representation | Type of feature extraction or representation employed (e.g., bag-of-words, TF-IDF, word embeddings, contextual embeddings). |
| Training and evaluation strategy | Description of data splitting or validation approach (e.g., train–test split, cross-validation), if reported. |
| **Model evaluation** |  |
| Performance metrics | Evaluation metrics reported for model performance (e.g., Accuracy, Precision, Recall, F1 score, AUC, correlation coefficients). |
| Best reported performance | Highest reported performance value(s) for the primary metric(s) used in each study. |
